# Effectiveness and Safety of Antibiotic Therapy Combined with NSAIDs or SAIDs in Osteomyelitis of the Oral and Maxillofacial Region: A Systematic Review

**DOI:** 10.3390/medicina61030499

**Published:** 2025-03-13

**Authors:** Heilyn Joanna Nils, Cristina Arce Recatalá, Cosimo Galletti, Javier Flores-Fraile

**Affiliations:** 1 Department of Stomatology and Surgery, Faculty of Medicine, University of Salamanca, 37008 Salamanca, Spain; j.flores@usal.es; 2 Department of Pharmacology, Valencia International University, 46002 Valencia, Spain; cristina.arce@professor.universidadviu.com; 3 Faculty of Medicine and Surgery, Kore University of Enna, 94100 Enna, Italy; cosimo.galletti@unikore.it

**Keywords:** osteomyelitis, antibiotics, NSAIDs, glucocorticoids, dentistry

## Abstract

*Background and Objectives:* Osteomyelitis is a progressive bone infection requiring a combination of antimicrobial and anti-inflammatory therapies. While antibiotics remain the cornerstone of treatment, the role of NSAIDs and steroidal anti-inflammatory drugs (SAIDs) in modulating inflammation and improving clinical outcomes warrants further investigation. This systematic review evaluates the effectiveness and safety of combined antibiotic and NSAID/SAID therapy in osteomyelitis, aligning treatment strategies with disease stage and pathogenesis. *Materials and Methods:* A systematic search was conducted in Web of Science, Scopus, and PubMed from July 2024 to November 2024, following PRISMA and CARE guidelines. The studies were selected based on detailed pharmacological data, treatment outcomes, and follow-up analysis. The risk of bias was assessed using the Critical Appraisal Skills Programmed (CASP) tool. Statistical reliability between coders was evaluated using Cohen’s kappa coefficient (κ = 0.636–0.909) and intra-class correlation coefficient (ICC = 1.0). *Results:* Four case studies, representing acute, chronic, recurrent, and SAPHO syndrome-associated osteomyelitis, demonstrated variable responses to combined therapy. Antibiotics alone were effective in acute cases, while NSAIDs/SAIDs significantly contributed to inflammatory control in chronic and immune-mediated osteomyelitis. Glucocorticoids (e.g., prednisolone, methylprednisolone) showed efficacy in reducing systemic inflammation, with no major adverse effects reported. The transition from intravenous to oral antibiotic therapy was observed in all cases, ensuring sustained infection control. *Conclusions:* This review highlights the critical role of NSAIDs/SAIDs in complementing antibiotic therapy, particularly in chronic and refractory osteomyelitis. Stage-specific pharmacological interventions improve treatment outcomes, and future research should explore bisphosphonates and immunomodulatory agents to refine therapeutic approaches. These findings reinforce the need for personalized osteomyelitis management based on pathogenesis, microbiology, and disease progression.

## 1. Introduction

Osteomyelitis is an infectious pathology of the bones. The term was coined by the French surgeon Auguste Nélaton in 1844 [1]. In 1852, Édouard-Pierre-Marie Chassaignac provided a detailed description of osteomyelitis, characterizing it as a pathology that originates from an inflammatory process within the bone tissue. By this time, the disease and its pathogenesis were already recognized, but Chassaignac’s work contributed to a more precise understanding of its underlying mechanisms.

The classification of osteomyelitis can be made according to various criteria (etiology, pathogenesis, etc.), but the most widely used is the Lew and Waldovgell classification into suppurative (acute and chronic), chronic sclerosing (focal and diffuse), and proliferative periostitis. Acute suppurative osteomyelitis presents intense pain, inflammation, and purulent discharge, while chronic osteomyelitis, although it shares characteristics, is milder and usually asymptomatic due to the encapsulation of the affected area. Focal sclerosing chronic osteomyelitis, the most common in young people, is asymptomatic and identified radiographically as a radiopaque lesion. Diffuse osteomyelitis, the most frequent variant in the elderly, manifests as a generalized bone sclerosis. Proliferative periostitis, or Garré osteomyelitis, is characterized by a thickening of the periosteum and formation of new bone, visible as “onion skin” on radiographs [2]. Osteomyelitis classification is critical for guiding clinical decision-making and tailoring appropriate therapeutic strategies. While traditional classifications categorize osteomyelitis based on etiology, pathogenesis, or clinical presentation, Marc Michael Baltensperger’s in (2004) [3] classification offers a pragmatic, clinically oriented approach by distinguishing between two major groups: destructive osteomyelitis and primary chronic osteomyelitis.

Destructive Osteomyelitis—This form of osteomyelitis follows a standard disease course, characterized by acute or chronic infection, progressive bone destruction, and the formation of sequestra (necrotic bone fragments). It often arises from hematogenous spread, contiguous infection, or direct inoculation. This type of osteomyelitis is generally amenable to antibiotic therapy and surgical debridement, with treatment tailored to the specific causative pathogen and the extent of bone involvement [4,5].

Primary Chronic Osteomyelitis—Unlike destructive osteomyelitis, primary chronic osteomyelitis is characterized by persistent, non-suppurative inflammation of the bone without a clear infectious etiology. This type of osteomyelitis often presents with sclerotic bone changes, and it is not amenable to standard antibiotic treatment. Instead, management focuses on anti-inflammatory therapy, including NSAIDs and immunomodulatory agents, and, in some cases, surgical intervention to control symptoms and prevent progression (Baltensperger et al., 2004; [3,6]).

Clinical manifestations may vary depending on the location, duration of infection, or the patient’s condition. Common symptoms include edema, fever, malaise, facial cellulitis, trismus, marked leukocytosis, decreased local blood flow, and pus formation, preceding osteonecrosis [4]. In addition, the severity of osteomyelitis may increase in the presence of underlying systemic conditions and/or sepsis [4,7].

Osteomyelitis is a bone infection that can be caused by bacteria, fungi, or other microorganisms. The most frequently associated pathogen with osteomyelitis is *Staphylococcus aureus,* followed by Staphylococcus epidermidis, and then *Enterobacteriaceae* and *Pseudomonas aeruginosa* [8]. However, in some cases, especially when there is some kind of compromise of the immune system or debilitating chronic diseases, the etiologic agents involved may be atypical bacteria. In addition, osteomyelitis can also be caused by fungal infections, including *actinomycetes*, *blastomycetes*, *coccidioides*, and *Cryptococcus neoformans* [4,7].

There are risk factors that compromise the patient’s immune response. These factors can be classified as systemic factors, which include advanced age, diabetes mellitus, hematological diseases, cancer, tuberculosis, obesity, HIV infection, malnutrition, anemia, syphilis, agranulocytosis, immunosuppressive treatments, such as chemotherapy and radiotherapy [9,10], and local factors, such as trauma, exposure to chemicals, electrocoagulation, irradiation, periodontal disease, tooth extractions, and pulp pathologies, such as caries and granulomas [11,12].

In dentistry, osteomyelitis occurs more frequently in the mandible compared to the maxilla. This condition is commonly a complication of odontogenic infections in immunocompromised individuals, affecting the osteon and, upon spread, compromising the periosteum [13]. Osteomyelitis of the maxilla is rare due to the extensive blood supply of the internal maxillary artery, whose anastomotic branches limit the spread of infection. Generally, this form of osteomyelitis arises from odontogenic infections in patients with immunologically predisposing conditions [14]. On the other hand, mandibular osteomyelitis is a severe inflammation of the bone that can be a consequence of dental infections, traumas which harbor pathogens in the tissues and result in bone fractures that communicate with the oral cavity, surgical procedures or systemic diseases. Their treatment is complex due to the inherent resistance of bone infections and the difficulty in achieving adequate penetration of antibiotics into the affected tissue [5].

The optimal treatment of osteomyelitis will depend on the antibiotic of choice and the route of administration, which will determine the antibiotic’s ability to reach the target bone tissue. A sample will be necessary to identify the pathogen causing osteomyelitis and thus be able to administer the most appropriate pharmacological treatment to the patient. Acute osteomyelitis is typically managed with antibiotics administered intravenously for 2 to 6 weeks, followed by oral therapy, while chronic cases require long-term antibiotic treatments (orally or through the use of locally acting controlled-release devices) [15].

From a dental perspective, in addition to surgical interventions such as debridement of necrotic tissue, extraction of the affected tooth, decortication, sequestrectomy and saucerization, antibiotic therapy is used. The most frequently used antibiotics in dentistry to treat osteomyelitis include Clindamycin, Metronidazole, Augmentin, Ciprofloxacin, Ampicillin, and Penicillin [16]. In cases of osteomyelitis caused by *Staphylococcus aureus*, the use of linezolid, daptomycin, or vancomycin is suggested. For hematogenous, post-traumatic or post-surgical osteomyelitis, the use of antibiotics effective against Gram-negative bacilli and staphylococci is recommended, highlighting third- and fourth-generation cephalosporins, as well as fluoroquinolones [17].

The treatment of osteomyelitis must be tailored to its pathogenesis, as the transition from acute infection to chronic biofilm-dominated disease necessitates distinct pharmacological strategies [4]. Acute osteomyelitis, driven by bacterial proliferation, requires early, high-dose IV antibiotics, with drug selection based on pathogen susceptibility and osseous penetration [8]. *Staphylococcus aureus*, particularly MRSA, dominates as the primary etiologic agent, necessitating vancomycin, linezolid, or daptomycin, while anaerobic and polymicrobial infections demand clindamycin or metronidazole [1,14].

In contrast, chronic osteomyelitis is characterized by biofilm formation and necrotic sequestration, significantly reducing antibiotic efficacy. Here, long-term antimicrobial suppression with rifampin-based regimens, combined with surgical debridement, is essential for infection control [18]. Fungal and atypical osteomyelitis, particularly in immunocompromised patients, necessitates prolonged azole or echinocandin therapy (Momodu and Savaliya).

NSAIDs and corticosteroids play a supportive role in attenuating immune-mediated bone resorption, particularly in non-suppurative osteomyelitis and SAPHO syndrome, but must be used judiciously to avoid suppressing host defenses in active infections (Demirci Yildirim and Sari, 2024) [6,19].

Thus, an evidence-based, pathogenesis-driven approach to osteomyelitis therapy is crucial, ensuring optimal antimicrobial selection and judicious inflammatory modulation to enhance treatment efficacy while minimizing therapeutic risks.

When establishing a diagnosis of osteomyelitis, the presence of musculoskeletal pain of sudden onset or progressive onset should be considered, particularly if associated with fever, malaise, lethargy, or irritability, although in immunocompromised adults these signs may be absent. Diagnostic suspicion increases in individuals with comorbidities such as poorly controlled diabetes mellitus, neuropathy, peripheral vascular disease, chronic wounds, recent trauma, sickle cell disease, orthopedic implants, or intravenous drug use. Physical examination can be decisive when findings such as erythema, soft tissue infections, bone pain, joint effusion, limited mobility, or exposed bone are identified. Bone probe testing is useful to exclude osteomyelitis in the low-risk diabetic foot [20,21].

Differential diagnosis includes soft tissue infections, gout, Charcot arthropathy, fractures, malignant neoplasms, bursitis, osteonecrosis, vaso-occlusive crises in patients with sickle cell disease, and SAPHO syndrome (synovitis, acne, pustular hyperostosis, and osteitis). Given the diagnostic uncertainty, additional evaluation that includes laboratory tests and imaging studies is necessary. The definitive diagnosis is established by a positive culture of bone biopsy. Polymerase chain reaction (PCR) facilitates rapid identification of pathogens, even in post-antibiotic cultures [22]. Although bone biopsy is the diagnostic standard, its applicability may be limited and some evidence suggests reserving it for specific cases, since the results do not always imply therapeutic changes [23].

The choice between antibiotics and NSAIDs in osteomyelitis treatment is fundamentally dependent on the stage of disease progression, the underlying pathogenesis, and the interplay between infection and inflammation.

Acute Osteomyelitis is primarily infection-driven, necessitating early, high-dose intravenous antibiotics to control bacterial proliferation before necrosis and sequestration occur (Lew and Waldvogel, 2004; Momodu and Savaliya, 2023) [4,18]. The selection of antibiotics depends on pathogen susceptibility and bone penetration, favoring beta-lactams, glycopeptides (vancomycin), or fluoroquinolones in biofilm-associated infections [8].

Chronic recurrent osteomyelitis presents with biofilm formation, bone necrosis, and immune dysregulation, reducing antibiotic efficacy. In such cases, NSAIDs and corticosteroids play a critical role in modulating inflammation and preventing bone destruction [24]. However, their immunosuppressive effects necessitate careful monitoring to prevent infection exacerbation [1].

SAPHO syndrome and non-suppurative osteomyelitis benefit significantly from NSAIDs (e.g., indomethacin, diclofenac) or corticosteroids due to their ability to suppress inflammatory osteolysis in sterile bone conditions [19,24].

In the oral cavity, mandibular osteomyelitis is a complex pathology in terms of its diagnosis, management, and effective resolution. Its multiple manifestations have given rise to various classifications, some of which represent subtypes of the same disease at different stages or with variable clinical expressions. These diagnoses are usually based on radiological characteristics, number of affected foci, age of the patient, presence or absence of purulent exudate, or recurrence of the condition. Radiologically, a mixed pattern of sclerosis and osteolysis is observed, with mandibular widening, periosteal reaction, and loss of definition in the cortico-medullary margins. In advanced stages, sclerosis predominates, with periosteal bone lamination and sequestrations, with an “onion skin” pattern being common in the subperiosteal bone formation, and with areas of osteosclerosis and irregular osteolysis [16].

In osteomyelitis, antibiotic therapy is accompanied by treatment to control pain and inflammation. Among the most commonly used analgesic and anti-inflammatory drugs in osteomyelitis are paracetamol, NSAIDs and SAIDs. A study was identified that addresses the use of methotrexate in the treatment of chronic recurrent multifocal mandibular osteomyelitis. On the other hand, Paim et al. recommend the administration of Methotrexate for refractory and painful cases. In the study, they documented three patients, where one of them showed clinical improvement after two months of combined treatment of NSAIDs (Rofecoxib 25 mg/day) and Methotrexate 20 mg/week [19]. However, although the study did not show adverse reactions, more evidence is required on the use of methotrexate in non-suppurative mandibular osteomyelitis, particularly on the safety profile since its side effects are life-threatening.

The decision to employ antibiotics versus NSAIDs—or their combination—depends on the interplay between infectious burden, host immune response, and the extent of bone involvement. Acute, infection-dominant osteomyelitis necessitates aggressive antibiotic therapy, whereas chronic or immune-mediated osteolytic conditions benefit from anti-inflammatory co-therapy to mitigate progressive bone damage. Understanding the dynamic shifts in osteomyelitis pathogenesis is therefore paramount in tailoring pharmacological interventions to optimize clinical outcomes while minimizing therapeutic risks.

Recurrent infection, persistent pain, and chronic inflammation are common challenges in the treatment of osteomyelitis. Therefore, it is hypothesized that the combination of glucocorticoids/NSAIDs and antibiotics administered as pretreatment is an effective and safe strategy for the management of osteomyelitis in dentistry, compared with its administration after dental treatment.

The aim of the present study is to evaluate the effectiveness and safety of the combination of antibiotics and NSAIDs or steroidal anti-inflammatory drugs in the management of osteomyelitis.

### 1.1. General Objective

Effectiveness and safety of treatment with antibiotics combined with NSAIDs and/or steroidal anti-inflammatory drugs administered to patients with osteomyelitis in dentistry.

### 1.2. Specific Objectives

1. To identify risk factors associated with both acute and chronic osteomyelitis in the context of maxillofacial surgery.
2. To identify the most commonly used treatments and administration routes in specific pharmacotherapy in patients with generalized, maxillary, or mandibular osteomyelitis.
3. To study the effectiveness and safety of treatment with antibiotics combined with NSAIDs and/or steroid anti-inflammatory drugs in patients with generalized osteomyelitis of the maxilla or mandible.

## 2. Methodology

Type of design: The study is a systematic review, not an interventional or observational study. To ensure clarity and accuracy, the methodology explicitly follows PRISMA and CARE guidelines, with PROSPERO registration, enhancing the study’s transparency and reproducibility. The study design is correctly defined as a systematic review of case reports, which is an established methodological approach to synthesizing clinical evidence in rare or complex conditions.

Research question: What is the effectiveness and safety of antibiotic therapy combined with NSAIDs or SAIDs in the management of osteomyelitis affecting the oral and maxillofacial region?

**Null Hypothesis** **(H_0_).** *The combination of antibiotic therapy with NSAIDs or SAIDs does not significantly improve treatment outcomes in osteomyelitis of the oral and maxillofacial region compared to antibiotic therapy alone.*

**Alternative Hypothesis** **(H_a_).** *The combination of antibiotic therapy with NSAIDs or SAIDs significantly improves treatment outcomes in osteomyelitis of the oral and maxillofacial region compared to antibiotic therapy alone.*

### 2.1. Search Strategy

#### Systematic Review Registration and Methodological Framework

This systematic review was designed and conducted following PRISMA and CARE guidelines to ensure methodological transparency and reproducibility. The study was prospectively registered in PROSPERO (CRD42024575611) to enhance credibility and adherence to best practices in systematic review methodology. To ensure the quality of the systematic review study design, the CARE (Case Report), and PRISMA (Preferred Reporting Item for Systematic Reviews and Meta-Analysis) guidelines were followed [25,26]. (Appendix A—Annex 1—PRISMA) (Annex 2—CARE).

A comprehensive search strategy was implemented across Web of Science, Scopus, and PubMed from July 2024 to November 2024, utilizing a predefined combination of Synonyms and truncation operators (*), Boolean (AND, OR, NOT) and position (“) Medical Subject Headings (MeSH) terms, and keyword variations to ensure exhaustive literature retrieval were used, adapted to each database (Table 1), following the search equations shown in (Table 2). In addition, we did not restrict the search in the databases by publication status or date, but by language (English, Spanish, Portuguese) and type of document (original article).

### 2.2. Eligibility Criteria for Studies

Randomized controlled trials (RCTs), cohort studies, case–control studies investigating antibiotic therapy in combination with NSAIDs or SAIDs for osteomyelitis were included in the systematic review. Please, see Table 3.

### 2.3. Data Extraction

The study selection followed a two-step process, comprising title/abstract screening and full-text assessment, conducted independently by two reviewers (HN, CA) to minimize selection bias. Discrepancies were resolved through discussion or adjudication by a third reviewer. Data extraction was standardized using a structured protocol, focusing on demographic characteristics, disease classification, treatment regimens, therapeutic response, and adverse effects a final decision was requested from a third author (JF-F). Full manuscripts of potential studies were obtained and reviewed for final inclusion and data extraction.

Following a coding book and a coding protocol previously established for the present systematic review, data extraction from the articles selected for this study was carried out by two independent coders (HN and CA). To ensure the reliability of the process, each coder performed the data extraction from the studies included in the present systematic review.

This systematic review excluded gray literature to ensure methodological rigor, scientific credibility, and data reliability. Only peer-reviewed studies from indexed databases (Web of Science, Scopus, PubMed) were included, as gray literature lacks standardized peer review, may introduce bias, and often present non-validated findings. While excluding gray literature minimizes data inconsistency, future studies may consider preprints and clinical trial registries to enhance comprehensiveness.

A structured coding book and coding protocol were developed to ensure systematic data extraction and inter-coder reliability. The coding book included predefined categories, such as demographics, disease classification, pharmacological regimens, clinical outcomes, and study quality indicators. Two independent coders (HN, CA) conducted data extraction, with Cohen’s kappa coefficient (κ = 0.636–0.909) and intra-class correlation coefficient (ICC = 1.0) confirming a high degree of inter-coder agreement. A third researcher (CG) was responsible for filtering and pre-screening eligible articles based on predefined inclusion criteria, ensuring the selection of high-quality, relevant studies before data extraction.

By implementing a rigorous coding framework and excluding non-peer-reviewed sources, this study enhances transparency, reproducibility, and the validity of its findings on osteomyelitis treatment strategies.

Discrepancies were resolved by consensus with a fourth author (J.F-F). The inter-coder reliability was good, with a mean kappa coefficient of 0.909 and mean intra-class correlations of 1. The criteria for resolving discrepancies included (1) cross-referencing the original study to verify extracted information, (2) evaluating the alignment of extracted data with the study objectives and inclusion criteria, and (3) adhering to predefined coding definitions and parameters to ensure standardization. 

The following data were collected from each study analyzed: descriptive variables of the studies (author, year, country, language, type of study, age, sex, pathology), descriptive outcome variables (pharmacological treatment, route of administration, patient follow-up), and risk of bias variables of the primary studies using the CASP (Critical Appraisal Skills Programmed).

### 2.4. Assessing the Risk of Bias in Primary Studies

All trials included in this study were assessed for risk of bias using the Critical Appraisal Skills Programmed (CASP) tools for detecting risk of bias in case studies or case series. The CASP tool was used to assess the risk of bias in case studies. This is a standardized guide designed for the analysis of clinical cases. It consists of 8 items covering essential aspects such as validity, quality and applicability of each case. It includes sections such as detailed description of patient information (age, gender, history), clinical findings, chronology of events, diagnosis, therapeutic interventions and results, as well as follow-up and patient perspective. Each item is evaluated with a yes or no, depending on whether or not the elements are present in the case study. Each item receives one point if it is present and zero points if it is absent. The total score (0–8 points) reflects the internal validity and quality of the study selected for this systematic review. In this study, each evaluator independently rated the 4 case studies using the CASP tool, and these evaluations were sent by email to be compared and agreed upon (Annex 3—CASP). Articles with a CASP score higher than 6 were considered eligible for inclusion in the present systematic review, whereas case studies with a score lower than 6 were excluded from the present systematic review. Discrepant scores between evaluators were discussed with a third author (JF-F) who made the final decision.

### 2.5. Statistical Study

The data extraction and study outcomes were prepared by two independent researchers, and then the reliability of the screening will be compared. To analyze the reliability and to interpret study results of the screening process, the free available statis-tical JASP 0.18.3.0 software is used. Cohen’s kappa coefficient was employed to assess the inter-rater agreement for categorical variables or small group, with values interpreted as follows: <0.20 (no agreement), 0.21–0.40 (poor agreement), 0.41–0.60 (moderate agreement), 0.61–0.80 (good agreement), and ≥0.8 (very good agreement). For continuous variables (such as age), the intra-class correlation coefficient (ICC) was calculated, with values interpreted as follows: <0.30 (no agreement), 0.31–0.50 (poor agreement), 0.51–0.70 (moderate agreement), 0.71–0.90 (good agreement), and ≥0.9 (very good agreement).

A Cohen’s kappa coefficient ranged from of 0.636 to 0.909 obtained in this study indicates very good agreement, demonstrating that the coders exhibited high consistency in their data extraction, thus minimizing potential biases. In addition, an ICC value of 1.0 observed in this study signifies perfect reliability, meaning that the extracted numerical data were consistent across coders without variation. These statistical methods validate the integrity of the data extraction process by confirming coder reliability and ensuring that the extraction data were systematically applied.

## 3. Results

### 3.1. Selection of Studies

The initial systematic search identified a total of 514 articles, distributed as follows: 169 from Web of Science, 67 from Scopus and 278 from PubMed. Duplicates and articles marked as ineligible were subsequently eliminated using the Zotero automation tools, which aims to filter out irrelevant documents or those that do not meet the basic inclusion criteria, thus optimizing the selection process. Additionally, a manual review was performed by title and abstract, resulting in the complete evaluation of 74 scientific articles.

Of these, 46 articles were excluded after a detailed evaluation, because they did not meet the eligibility criteria, such as lack of specific information on the methodology applied, absence of data relevant to the focus of the study or lack of clinical representativeness.

**Figure 1:**
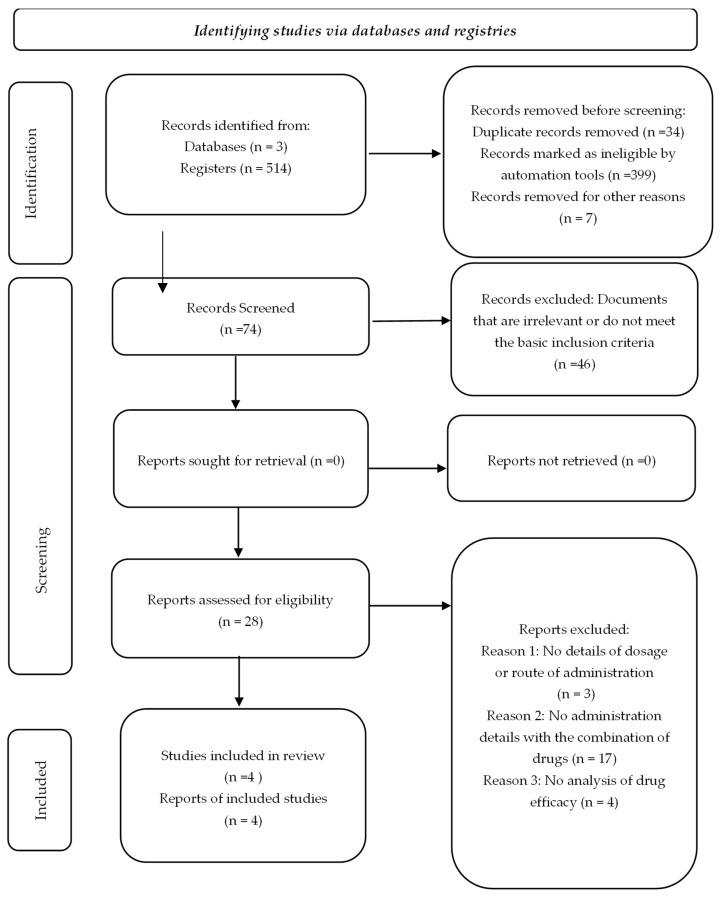
PRISMA 2020 flowchart including searches only in databases and registries.

The flow chart illustrating the process of searching for and selecting studies is presented in Figure 1 and Figure S1. Finally, four studies were selected that, although initially classified as controlled clinical trials (RCTs), corresponded to the case reports, highlighting the importance of a thorough systematic review.

### 3.2. Results of Individual Studies

The study population includes patients diagnosed with suppurative osteomyelitis (acute and chronic), chronic sclerosing osteomyelitis (focal and diffuse), or proliferative periostitis of any age and sex. The diagnosis of osteomyelitis depends on clinical symptoms, including fever, pain, and swelling over the affected bone. The use of laboratory tests, including positive blood culture, ESR (erythrocyte sedimentation rate) and CRP (C-reactive protein), as well as diagnostic imaging, such as MRI, CT, nuclear magnetic imaging, ultrasound or X-ray, and biopsies, supports the diagnosis: (a) clinical signs of osteomyelitis, (b) positive blood cultures, (c) radiological support.

The differential diagnosis of osteomyelitis encompasses a broad spectrum of pathologies that must be carefully considered during the clinical evaluation of a patient with suspected bone infection. Conditions that may mimic or be confused with osteomyelitis include Charcot arthropathy, which is common in diabetic patients; SAPHO syndrome (synovitis, acne, pustulosis, hyperostosis, and osteitis); various forms of arthritis, including rheumatoid arthritis; metastatic bone disease; pathologic and stress fractures; gout; avascular necrosis of bone; bursitis; and vaso-occlusive pain crises associated with sickle cell anemia [18].

The total sample consisted of 4 participants, 1 participant per case report.

The classification system applied in this review aligns with the Marc Michael Baltensperger’s (2004) [3] framework, which categorizes osteomyelitis into two primary subtypes: destructive osteomyelitis and primary chronic osteomyelitis [3].

The case report by Kudva et al. (2019) [27] presented a 32-year-old man who presented to the clinic with the following symptomatology: dull pain (approximately 6 weeks), swelling in the left jaw, and a reduction in mouth opening. After a clinical and laboratory evaluation, he was diagnosed with a recurrent form of mandibular osteomyelitis. Complementary studies were performed that showed an increase in the erythrocyte sedimentation rate (ESR) and leukocyte count, in addition to elevated levels of osteoprotegerins (OPG). The X-ray of the mandible showed increased opacity with trabecular alterations in the affected region, while the MRI showed a hypointense signal in T1WI sequences and hyperintense in T2WI and STIR, together with a cortical thickening that compromised the left hemimandible. In this case, no surgical intervention was performed other than an intraoral biopsy under local anesthesia and intravenous sedation in order to confirm the diagnosis of osteomyelitis.

The patient required hospitalization for a recurrent chronic inflammatory infection, where he was administered the following medications intravenously: Augmentin (amoxicillin 500 mg with clavulanic acid potassium 125 mg, three times a day for one week) and Clindamycin (300 mg, twice a day for one week) combined with NSAIDs (without specification of the NSAID used). After discharge, the patient continued with oral antibiotics, NSAIDs, and prednisolone (40–60 mg, once a day for one week). This drug combination was effective, as it produced stabilization of the clinical picture, resolution of pain, and control of the infection without the need for major surgery (Table 4). The case report by Kudva et al. (2019) [27] describes recurrent chronic osteomyelitis of the mandible, a form of destructive osteomyelitis managed with a combination of antibiotics, NSAIDs, and corticosteroids, resulting in successful clinical stabilization.

In the case report by Lambade et al. (2013) [28], a 35-year-old woman presented to the clinic with the following symptoms: difficulty opening the mouth and subauricular purulent discharge on the left side, symptoms that had persisted for 2 to 3 months. The history revealed no relevant antecedents to explain the clinical picture, and the intraoral examination revealed no pathological findings in the teeth or adjacent tissues. Upon further investigation, the presence of an ectopic mandibular third molar was identified, which had caused the formation of an extraoral sinus and a subauricular scar, associated with osteomyelitis of the mandibular condyle. Finally, chronic suppurative osteomyelitis of the mandibular condyle was diagnosed. The surgical intervention consisted of the exposure of the affected condyle through an extraoral preauricular incision. Extraction of the ectopic third molar was performed along with infected granulation tissue and small bone fragments from the buccal cortex. The procedure included careful curettage to avoid fractures of the mandibular condylar process, preserving most of the condylar structure and ensuring that mandibular function was not compromised.

Postoperative pharmacotherapeutic management included a 7-day regimen of oral anti-inflammatory antibiotics and analgesics: amoxicillin 500 mg/clavulanic acid 125 mg three times daily; metronidazole 400 mg three times daily; and diclofenac sodium 50 mg twice daily. This drug combination was effective, achieving resolution of symptoms.

The patient did not show mandibular deviation or functional alterations, and the preoperative occlusion remained unchanged at 18 months of follow-up (Table 4). The case by Lambade et al. (2013) [28] details chronic suppurative osteomyelitis of the mandibular condyle, necessitating surgical curettage and long-term antibiotic therapy, consistent with destructive osteomyelitis.

In the case report by Roldán et al. (2001) [29], a 26-year-old man was described with a diagnosis of SAPHO syndrome (synovitis, acne, pustulosis, hyperostosis, and osteitis). The patient was presented with severe jaw pain, edema, and limited mouth opening. Bone scintigraphy revealed diffuse involvement of the entire mandible, along with additional lesions in the sternum, ileum, greater and lesser trochanters of the left femur, and left distal tibia. Propionibacterium was isolated on four occasions and Staphylococcus epidermidis was isolated from samples taken from the acne and from a hip lesion. The clinical picture began with severe acne conglobata, followed by aseptic arthritis in the left knee and nonsuppurative osteomyelitis of the mandible. During episodes of exacerbation of osteoarticular symptoms, the acne also worsened. In addition, the patient experienced increased liver enzyme levels and an immunologic response associated with non-A, non-B hepatitis. Painful osteomyelitis of the left hip caused difficulty in walking and became the patient’s main complaint. The diagnosis of mandibular osteomyelitis was established by a combination of clinical evaluation and advanced imaging techniques. Bone scintigraphy was essential, as it showed diffuse involvement throughout the mandible, confirming the presence of a chronic inflammatory process in the bone. This technique, which uses radiopharmaceuticals to detect areas of high bone metabolic activity, allowed the identification of both mandibular osteomyelitis and additional lesions in other regions of the skeleton, characterizing the systemic scope of SAPHO syndrome. The clinical correlation included intense mandibular pain, edema, and limitation in mouth opening, classic symptoms of osteomyelitis in this region. In addition, the isolation of Propionibacterium and Staphylococcus epidermidis from acne samples and hip lesions supported the infectious component associated with the systemic condition. The treatment included mandibular decortication procedures with administration, placement of gentamicin-PMMA beads, and hyperbaric oxygen therapy sessions.

Regarding pharmacotherapy, in the acute phases of the disease, the patient received clindamycin (two years) intravenously. This was followed by oral treatment with amoxicillin-clavulanate (500 mg/125 mg, 3 times a day for 7 days), prednisolone (5 mg, once a day), minocycline (50 mg, twice a day), and isotretinoin (10 mg, twice a day) for the treatment of acne during the three-year period. This drug combination was partially effective, as there was significant remission, although with persistence of systemic osteoarticular symptoms associated with SAPHO syndrome (Table 4). Roldán et al. (2001) [29] reported SAPHO syndrome-associated osteomyelitis, a primary chronic osteomyelitis variant, requiring long-term immunomodulation alongside antibiotic therapy.

In the case report by Holden et al. (2005) [24], a 27-year-old woman was reported with the following clinical history: 13 years of recurrent episodes of low back and right leg pain, associated with elevated inflammatory markers. The back pain began after a fall at age 14, although there were no associated penetrating injuries. Initial radiological and scintigraphy studies identified lesions compatible with osteomyelitis in the sacrum, distal femur, and one rib. Although cultures obtained from the sacral aspirate were negative, a presumptive diagnosis of osteomyelitis was made.

The patient was treated with multiple courses of intravenous and oral antibiotics. However, over the course of three years, she continued to experience recurrent episodes of low back and thigh pain, accompanied by an elevated erythrocyte sedimentation rate (ESR) of 114 mm/h and a C-reactive protein (CRP) of 97 mg/L. Surgical excision of the presumed lesion on the right distal femur was performed. Postoperative management included a combination of vancomycin, meropenem, doxycycline, and ciprofloxacin. This therapeutic approach sought to cover a broad spectrum of pathogens, including resistant Gram-positive, anaerobic, and Gram-negative organisms, achieving a significant decrease in inflammatory markers, such as CRP (97 mg/L to normal levels) and ESR (114 mm/h). However, months later, symptoms resurged, with a new lesion identified on the right tibia by MRI, with an elevated CRP level of 109 mg/L. During an exacerbation, a new cycle of azithromycin was started, and 1 g of methylprednisolone was administered for two consecutive days. This treatment resulted in remarkable improvement, with normalization of CRP levels to less than 8 mg/L and resolution of the tibial lesion within two days.

This case study highlights a remarkable response to the use of corticosteroids, reflected by the rapid decrease in pain, normalization of inflammatory markers, and resolution of lesions evidenced by MRI (Table 4). Holden et al. (2005) [24] presented chronic recurrent multifocal osteomyelitis, which demonstrated significant responsiveness to corticosteroid therapy, highlighting the immune-mediated nature of primary chronic osteomyelitis.

Although oral osteomyelitis is not mentioned in this case report, the observed management combination of broad-spectrum antibiotics and corticosteroids could be extrapolated to scenarios of mandibular or maxillary osteomyelitis, given the complexity of inflammatory control and eradication of bone infections.

### 3.3. Rationale for the Selection of the Four Case Studies

The selection of the four case studies was based on a rigorous methodological framework to ensure a representative and clinically relevant analysis of pharmacological strategies in osteomyelitis treatment. The key criteria guiding selection were as follows.

Diversity in Clinical Presentation—The cases included acute, chronic, recurrent, and SAPHO syndrome-associated osteomyelitis, ensuring a broad spectrum of disease manifestations.

Comprehensive Pharmacological Data—Only cases with detailed documentation of antibiotic regimens, NSAID/SAID co-administration, dosing, and duration were included, allowing for a systematic assessment of treatment efficacy.

Outcome Evaluation—Cases provided robust follow-up data, including symptom resolution, radiological findings, and recurrence rates, to assess both short- and long-term therapeutic impact.

Integration of Surgical and Non-Surgical Approaches—The selection included both surgically managed and pharmacologically treated cases, enabling a comparative evaluation of medical versus combined treatment strategies.

Clinical Complexity and Comorbidities—The inclusion of a SAPHO syndrome-associated case highlights the challenges of osteomyelitis in systemic inflammatory disorders, while recurrent and post-traumatic cases illustrate the difficulties in long-term disease control.

This structured selection approach ensures that the study provides a comprehensive and methodologically sound evaluation of NSAID and SAID efficacy in osteomyelitis management, balancing clinical variability, treatment outcomes, and therapeutic strategies.

### 3.4. Identify Risk Factors Associated with Both Acute and Chronic Osteomyelitis in the Context of Maxillofacial and/or General Surgery

Based on the four case reports and reviews presented, multiple risk factors are identified that predispose patients to the onset, development, and recurrence of osteomyelitis. These include anatomical, microbiological, and immunological aspects, as well as limitations in therapeutic strategies.

First, regarding the observed anatomical factors, we can see in the case report by Lambade et al. (2013) [28] structural alterations of an ectopic mandibular third molar, which acted as the initial focus of infection and progressed to osteomyelitis of the mandibular condyle. This case highlights how the presence of abnormal anatomical structures can facilitate bacterial colonization and chronic inflammation. In the case study by Holden et al. (2005) [24], a lumbar trauma in adolescence was referred to as a trigger for recurrent multifocal osteomyelitis in the sacrum and femur, evidencing the vulnerability of bone tissue after previous injuries even without penetrating exposure.

Second, in the case report by Roldán et al. (2001) [29], the isolation of Propionibacterium and Staphylococcus epidermidis in multifocal mandibular and bone lesions shows the central role of opportunistic bacteria in patients with comorbidities such as acne conglobata. The chronicity of the infection and its resistance to conventional treatments reflect the difficulty in eradicating microorganisms well adapted to specific environments.

Third, the case report by Kudva et al. (2019) [27] showed that prolonged corticosteroid treatments, although effective in controlling systemic inflammation, may compromise the immune system’s ability to manage active infections. In patients with osteomyelitic recurrence, this immunosuppression, combined with the chronic nature of the pathology, exacerbates the difficulty in controlling the disease.

Fourth, it was shown that another risk factor is the association of osteomyelitis with SAPHO syndrome (synovitis, acne, pustulosis, hyperostosis and osteitis), evidenced in the case study of Roldán et al. (2001) [29], since systemic inflammatory disorders predispose to the dissemination of bone infections.

Finally, the case report by Kudva et al. [27] and Holden et al. [24] highlighted the delay in identifying the initial focus, which led to clinical complications. Furthermore, the case report also showed the importance of patient follow-up in order to prevent relapses.

These findings highlight the complexity of managing osteomyelitis, with early identification and mitigation of risk factors being crucial to prevent its progression. They also reinforce the need for comprehensive approaches combining precise surgical interventions, personalized pharmacological treatments, and constant clinical monitoring.

### 3.5. Study of the Effectiveness and Safety of Treatment with Antibiotics Combined with NSAIDs and/or Steroid Anti-Inflammatory Drugs in Patients with Osteomyelitis of the Maxilla or Mandible

Here, we analyze the efficacy and safety of combined antibiotic pharmacotherapy with NSAIDs or glucocorticoids in patients with generalized maxillary or mandibular osteomyelitis, evaluating the ability to reduce infection, inflammation, pain. The objective is to provide knowledge in optimizing the management of osteomyelitis in the context of maxillofacial/general surgery.

For the management of acute bacterial infection in the case report by Kudva et al. 2019 [27] and Roldán et al. 2001 [29], a combination of Amoxicillin-clavulanate and clindamycin was used.

The combination of amoxicillin-clavulanate, which covers Gram-positive, Gram-negative and anaerobic bacteria, and clindamycin, effective against resistant anaerobic and Gram-positive bacteria, allows a broad spectrum of action to treat mandibular infections. The therapy effectively controlled the infection and inflammation without the need for extensive surgical interventions. In addition, treatment was accompanied by NSAIDs and prednisolone. NSAIDs control pain and acute inflammation, while corticosteroids such as prednisolone are widely used drugs to modulate the inflammatory response and reduce systemic inflammation. This therapeutic approach showed effective control of infection and inflammation without the need for surgical intervention in the patient who presented the diagnosis of recurrent mandibular osteomyelitis (Kudva et al., 2019) [27]. The stabilization of the clinical picture and the absence of relapses during follow-up suggest that the combination optimized the systemic management of the inflammatory process, highlighting the usefulness of glucocorticoids in modulating the chronic inflammatory response. However, in the patient with mandibular osteomyelitis associated with SAPHO syndrome (Roldán et al. 2001) [29], the combination of long-term antibiotics (amoxicillin-clavulanate, clindamycin) with prednisolone and targeted therapies (minocycline, isotretinoin) showed partial efficacy. Although there was a significant remission of the mandibular lesions and temporary control of pain, the persistence of systemic and osteoarticular symptoms shows the complexity of this chronic inflammatory pathology. The efficacy of glucocorticoids was clear for pain management, but not sufficient to achieve complete resolution of the condition.

In the case report by Lambade et al. [28], they used the combination of the antibiotics Amoxicillin-clavulanate and metronidazole. Metronidazole is an antibiotic widely used in the prevention of postoperative anaerobic bacterial infections (such as osteomyelitis). It was accompanied by the use of diclofenac sodium to control postoperative pain and reduce inflammation after surgery. This pharmacological combination was effective as a complement to surgical management, ensuring functional recovery and absence of recurrences in the patient diagnosed with chronic suppurative osteomyelitis of the mandibular condyle. Although therapeutic success was mainly due to surgical intervention, the pharmacological combination contributed to controlling postoperative inflammation and preventing residual infections. Long-term follow-up confirmed the absence of recurrence or mandibular dysfunction, supporting the efficacy of this approach in cases where surgical resolution is possible.

Finally, the case report of Holden et al. (2005) [24] combined prolonged courses of antibiotics, surgical excision, and the use of methylprednisolone to address recurrent multifocal osteomyelitis. In this case, of recurrent sacral osteomyelitis, initial treatment with antibiotics failed to achieve adequate control of the disease. However, the combination of intravenous antibiotics and methylprednisolone was highly effective during an acute exacerbation, achieving rapid normalization of inflammatory markers (CRP and ESR) and resolution of lesions evidenced by magnetic resonance imaging. This result highlights the critical role of corticosteroids in modulating severe inflammation and improving clinical response when combined with targeted antimicrobial therapy.

These results suggest that surgical treatments combined with antibiotics showed greater effectiveness in focal cases, such as those reported by Lambade et al., while the use of corticosteroids, as in Holden et al., was key in complex pathologies with systemic inflammatory components. On the other hand, the cases of Kudva et al. and Roldán et al. highlight how appropriate pharmacological interventions may be sufficient or partial, depending on the complexity of the underlying clinical condition.

From a safety perspective, no major adverse effects directly related to NSAIDs or SAIDs were reported in the cases reviewed. However, it is important to remember that all drugs have side effects, so the choice of treatment should consider the patient’s specific conditions to minimize unwanted effects as much as possible. In addition, prolonged use of glucocorticoids, as in the cases of Roldán et al. and Kudva et al., may be associated with known risks, such as immunosuppression, osteoporosis, or metabolic alterations, although these were not documented in detail.

On the other hand, the extended use of antibiotics poses potential risks of bacterial resistance and dysbiosis, although no specific complications were mentioned in the studies.

### 3.6. Identify the Most Commonly Used Route of Administration in Specific Pharmacotherapy in Patients with Maxillary or Mandibular Osteomyelitis

From the review of the four case reports previously discussed, it has been shown that, in the pharmacotherapeutic management of osteomyelitis, particularly in cases of generalized extension in the maxilla and/or mandible, the most commonly used routes of administration of antimicrobial agents are the intravenous *(IV*) route and the oral route.

*IV* is the initial route of choice in the acute phase of infection, as it guarantees 100% bioavailability and constant therapeutic concentrations of the antibiotic in the circulatory system and in the affected bone tissue. This modality allows for a rapid pharmacodynamic effect, as it does not involve absorption, being the best therapeutic option in the administration of antibiotics that require high doses and urgency. However, the *IV* route requires administration by specialized health personnel, which limits the autonomy of the patient.

On the other hand, the oral route of administration is usually reserved for the continuity phase after control of the acute infection, or in patients with stable evolution, favoring prolonged administration and improving patient adherence to the therapeutic regimen. This strategy is particularly useful in the context of chronic infections or when outpatient therapy is required. In certain circumstances, the direct administration of antimicrobial agents at the site of infection using impregnated spacers or specific antimicrobial dressings is considered advantageous. This route optimizes the concentration of the drug at the infectious site, limiting systemic exposure and reducing the risk of adverse effects. However, this is a rarely used route of administration in patients with generalized osteomyelitis [30,31,32,33].

The study of osteomyelitis cases shows that the administration strategies of antibiotic therapy, combined with NSAIDs or corticosteroids, are integrated and combined according to the phase of development and evolution of the disease as well as the clinical severity of osteomyelitis, generally starting with the intravenous route and progressing to oral therapy to ensure complete resolution of the infection and minimizing the probability of recurrences.

## 4. Discussion

The findings of this systematic review suggest partial support for the alternative hypothesis (H_a_), while not entirely rejecting the null hypothesis (H_0_) due to variability in treatment response across different osteomyelitis subtypes. Evidence supports the alternative hypothesis (H_a_).

Clinical outcomes indicate improved symptom control with NSAID/SAID co-therapy. The analysis of acute, chronic, recurrent, and SAPHO syndrome-associated osteomyelitis cases demonstrates that NSAIDs and SAIDs play a crucial role in modulating inflammatory osteolysis and symptom severity, particularly in chronic and immune-mediated cases.

Glucocorticoids (e.g., prednisolone, methylprednisolone) exhibited notable efficacy in reducing inflammatory markers, pain levels, and radiographic progression in non-suppurative osteomyelitis. NSAIDs and SAIDs were adjuncts to antibiotics in chronic osteomyelitis. While antibiotic therapy alone was sufficient for infection resolution in acute osteomyelitis, chronic and recurrent cases demonstrated significant therapeutic benefits when NSAIDs or SAIDs were incorporated.

Steroid therapy improved treatment outcomes in SAPHO syndrome-associated osteomyelitis, supporting the role of anti-inflammatory agents in osteomyelitis subtypes with immune dysregulation.

### 4.1. Limitations and Considerations Supporting the Null Hypothesis (H_0_)

Lack of statistically significant comparative data: The review synthesizes case reports rather than controlled clinical trials, limiting the ability to quantify the extent of therapeutic benefit provided by NSAIDs/SAIDs.

The absence of a direct comparative cohort without NSAID/SAID therapy prevents definitive conclusions regarding the magnitude of impact.

Potential risks of NSAID/SAID therapy: While beneficial in chronic and immune-mediated osteomyelitis, NSAID/SAID use requires careful monitoring due to immunosuppressive effects, which may, in some cases, delay bacterial clearance or exacerbate infection risks in inadequately controlled cases.

The study findings lean toward supporting the alternative hypothesis (H_a_), particularly in chronic and inflammatory-driven osteomyelitis, where NSAIDs and SAIDs enhance symptom control and treatment outcomes. However, due to methodological limitations, absence of randomized data, and inherent variability in osteomyelitis pathophysiology, the null hypothesis (H_0_) cannot be entirely refuted.

Future studies, including prospective controlled trials, are essential to quantify the comparative efficacy of NSAID/SAID therapy, refine treatment protocols, and establish stage-specific therapeutic guidelines in osteomyelitis of the oral and maxillofacial region. Maxillofacial space infections (MSI) are mostly of odontogenic origin, followed by lymphadenitis and trauma, with the former being the most prevalent [34]. The use of modern antibiotics has significantly decreased the mortality associated with these infections [35]. Common symptoms of MSI include edema, trismus, pain, odynophagia and dysphagia, all derived from the inflammatory response to the infection, which can aggravate the symptoms. Corticosteroids, known for their ability to mitigate edema and inflammation in head and neck pathologies [36], are postulated as adjuvants in the management of MSI, by enhancing the immune response in combination with antibiotics [37]. These drugs inhibit the transcription of proinflammatory mediators in endothelial cells, reducing pharyngeal inflammation and pain [38]. Lamkin and Portt (2006) documented a remarkable synergy between corticosteroids and antibiotics in the treatment of craniofacial infections [39]. Although some fear that systemic corticosteroids may suppress the immune response, a short course of dexamethasone (three IV doses of 8 mg every 8 h) is unlikely to induce immunosuppression.

Despite limited evidence, the combination of corticosteroids and antibiotics in the treatment of MSI shows promising results [40].

The combination of antibiotics with nonsteroidal anti-inflammatory drugs (NSAIDs) or glucocorticoids (SAIDs) in the four cases analyzed shows variable results depending on the etiology, clinical management, and evolution of the pathology, which demonstrates both the strengths and limitations of this multidimensional therapeutic approach in the treatment of osteomyelitis.

Regarding the risk factors predisposed to osteomyelitis, the analyzed cases highlight a history of chronic infections, previous traumas, and systemic conditions such as SAPHO syndrome. These conditions predispose to the development of chronic or recurrent osteomyelitis. In fact, in the study by Roldán et al. (2001) [29], SAPHO syndrome represented a key underlying factor that exacerbated infections and osteoarticular complications. Similarly, Holden et al. (2005) [24] highlight how early traumas can evolve into chronic conditions when not managed in a timely manner.

Regarding combined treatment with antibiotics and NSAIDs or glucocorticoids and predominant routes of administration, a preference for intravenous antibiotics was observed in the acute phases, with a transition to oral administration for maintenance. Kudva et al. [27] and Lambade et al. [28] used amoxicillin-clavulanate in combination with other antibiotics, such as clindamycin or metronidazole, showing an effective reduction in infection. However, surgical treatments, as demonstrated by Lambade et al. and Roldán et al., proved to be essential in cases where the infection had significantly advanced. The use of corticosteroids, as in Holden et al. [24] and Kudva et al. [27] was effective in controlling inflammation, although their safety should be carefully evaluated, especially in patients with comorbidities.

From a comprehensive view, in three of the four cases, the combination of antibiotics with NSAIDs or SAIDs was effective in controlling infection and inflammation, either primarily (Kudva, Lambade) or as part of combined management with surgery (Roldán, Lambade). However, the case of Holden et al. highlights the need for therapeutic adjustments in chronic or recurrent infections. Glucocorticoids, used in two cases (Kudva and Holden), prednisolone and methylprednisolone, showed a significant impact on the resolution of systemic inflammation, especially in acute conditions or with elevated inflammatory markers, while NSAIDs were limited to symptomatic and postoperative control.

Despite the positive results, these cases underline the importance of a personalized approach, considering the etiology, chronicity, and possible complications associated with prolonged use of glucocorticoids or antibiotics, such as immunosuppression, bacterial resistance or systemic toxicity. The combination of these therapies should be carefully monitored to optimize results and minimize risks.

### 4.2. Adverse Effects Observed with the Combination of Antibiotics, NSAIDs and Glucocorticoids

Regarding antibiotic therapy, prolonged antibiotic cycles, as in the cases of Roldán et al. [29] and Holden et al. [24] may have generated risks of dysbiosis, bacterial resistance, and liver toxicity. In the case of Roldán et al. [29] an increase in liver enzymes was documented. Although NSAIDs are useful for controlling pain and inflammation, their prolonged use may increase the risk of gastrointestinal and renal complications; however, these were not directly reported in the cases. Finally, with the administration of glucocorticoids, as in the cases of Roldán et al. [29] and Holden et al. [24] the use of prednisolone and methylprednisolone was effective in controlling inflammation. However, prolonged glucocorticoid therapy may be associated with immunosuppression, osteoporosis, and metabolic alterations, especially in the long-term management of SAPHO syndrome. It should be noted that no adverse reactions were documented in the study.

Together, the combination of these drugs has been shown to be effective in controlling infection and inflammation, although it requires careful monitoring to prevent adverse effects associated with prolonged treatment.

### 4.3. Adjunctive Therapies in Osteomyelitis

Beyond conventional antimicrobial and surgical management, bisphosphonates and immune-correcting drugs are emerging as adjunctive treatments for osteomyelitis, particularly in chronic and non-suppurative forms.

Bisphosphonates inhibit osteoclast-mediated bone resorption, making them beneficial in chronic sclerosing osteomyelitis and SAPHO syndrome, where pathological bone remodeling is a major concern [6].

Immunomodulator drugs, such as TNF-α inhibitors, interleukin blockers (IL-1, IL-6), and methotrexate, have been explored in autoimmune-related osteomyelitis, helping to regulate dysregulated inflammation and reduce bone destruction [19].

Incorporating these agents into osteomyelitis treatment paradigms may enhance therapeutic outcomes in refractory or immune-mediated cases, offering a more targeted approach beyond conventional antibiotics and anti-inflammatories.

### 4.4. Limitations

This study evaluates the efficacy and safety of using nonsteroidal anti-inflammatory drugs (NSAIDs) and steroid anti-inflammatory drugs (SAIDs) as adjuvants to antibiotic therapy in the treatment of osteomyelitis; however, it has important limitations that affect its scope and validity. Firstly, the availability of specific scientific literature for the population over 18 years of age is remarkably limited, which considerably restricts the generalizability of the results and affects the representativeness of the sample. This lack of specific studies in this age group introduces a limitation in external validity, making it difficult to extrapolate the findings to other demographic subgroups of the adult population.

Furthermore, the studies analyzed show a lack of uniformity in dosage, administration time and specific types of NSAIDs and SAIDs used, which increases variability and makes it difficult to compare results between different studies. Osteomyelitis itself encompasses a wide spectrum of clinical presentations and etiologies, which makes it difficult to form homogeneous groups in the studies.

The small sample size, heterogeneity in patient health status, sources of infection, and comorbidities represent additional confounding factors that limit the study’s ability to isolate the specific impact of NSAIDs and SAIDs as adjuvants.

Furthermore, potential adverse reactions, especially in long-term or high-dose treatments, are often underestimated in short-term studies, which restricts the com-prehensive evaluation of the safety profiles of these drugs. This lack of long-term in-formation underscores the need for prolonged studies with diverse demographic representation, as well as standardized protocols, in order to more accurately establish the efficacy and safety of the use of NSAIDs and SAIDs as adjuvants in the management of osteomyelitis.

In summary, although the case studies analyzed offer valuable information on the management of pharmacotherapy in cases of osteomyelitis, the limitations noted highlight the need for more rigorous future research and long-term follow-up of pharmaco-logical treatments to assess effectiveness and safety.

### 4.5. Future Perspectives

Future research on the combined use of antibiotics and NSAIDs or SAIDs in osteomyelitis could significantly benefit from a multidisciplinary approach including genetic profiling and molecular insights. A deeper understanding of genetic predispositions and molecular pathways involved in inflammation, immune response, and bone metabolism may allow for more personalized and effective therapeutic strategies.

By identifying specific genetic markers associated with the regulation of inflammation, immune response variability, and drug metabolism, future studies could target treatments to individuals who are most likely to benefit from adjunctive antibiotic therapy with NSAIDs or SAIDs, thereby increasing therapeutic efficacy and minimizing adverse effects [41].

Genetic studies in osteomyelitis have suggested that certain polymorphisms in genes associated with immune modulation, such as those encoding cytokines (e.g., IL-1, IL-6, TNF-α), may affect disease susceptibility, progression, and response to treatment [6]. Integrating this knowledge into clinical practice may allow clinicians to predict patient responses to NSAIDs and SAIDs more accurately, ultimately paving the way for personalized medicine approaches. Pharmacogenomics, which examines how genetic differences affect an individual’s response to medications, could further refine dosing protocols and the choice of specific NSAIDs or SAIDs, particularly in relation to their metabolism and potential for side effects. For example, genetic variations in cyto-chrome P450 enzymes, which metabolize many NSAIDs, could influence drug efficacy and safety, highlighting the need for genotype-guided treatment adjustments [41].

Another promising avenue for future research involves exploring gene therapy and molecular inhibitors that specifically target inflammatory pathways relevant to osteomyelitis. Advances in CRISPR and other gene editing technologies could one day enable targeted modification of genes associated with chronic inflammation and bone degeneration, potentially offering a curative approach that complements antibiotic and anti-inflammatory treatments [42]. This approach could minimize reliance on long-term NSAID or SAID therapy and their associated risks, providing a more sustainable solution for managing osteomyelitis.

In summary, incorporating genetic knowledge into the treatment of osteomyelitis could transform current therapeutic paradigms by improving precision, reducing side effects, and improving long-term outcomes.

Future research should prioritize longitudinal studies with genetic and pharmacogenomic analyses to optimize and personalize the use of NSAIDs and SAIDs, moving beyond the traditional one-size-fits-all model toward a more personalized and efficient therapeutic strategy.

## 5. Conclusions

1. The most relevant risk factors in the pharmacological management of osteomyelitis include a history of chronic infections, previous traumas, and systemic conditions, such as SAPHO syndrome, which must be evaluated comprehensively to adapt the pharmacological treatment.
2. Pharmacotherapy of osteomyelitis involved the initial administration of intravenous antibiotics (such as amoxicillin-clavulanate, clindamycin, vancomycin, or meropenem) in the acute phases, with a change to oral routes for the maintenance and control of bacterial infection. In advanced or complex cases of pathology, dental surgery complemented pharmacological management.
3. The combination of antibiotics with NSAIDs/glucocorticoids (SAID) is, in general, an effective therapeutic strategy for the management of osteomyelitis in various clinical presentations. This approach has demonstrated its ability to control infection, modulate inflammation, and improve clinical outcomes in most patients studied. However, its efficacy and safety depend on specific factors, such as the etiology of the disease, the chronicity of the condition, and the individual response to treatment.

Taken together, the reviewed data support the efficacy of combining antibiotics with NSAIDs and SAIDs for the management of osteomyelitis in specific contexts. However, therapeutic success depends on a personalized approach that considers the individual characteristics of each case and incorporates careful monitoring to minimize the risks associated with prolonged treatment. It is crucial that future studies include detailed monitoring of potential adverse effects and optimize treatment regimens to maximize clinical benefits in similar populations.

## Figures and Tables

**Table 1:** Breakdown of the general objective. Truncation operators (*) and Position operators (“).

Pathology	Patient	Pharmacotherapy	Administration Route
Osteomyelitis “Chronic osteomyelitis” “Acute osteomyelitis” Osteomyelitis “Chronic Osteomyelitis” “Acute Osteomyelitis”	Adult*	Pharmacological treatment* Drug* Medication* Treatment* Antibiotics Antibiotic therapy NSAIDs Analgesic* Nonsteroidal anti-inflammatory NSAID* Non-steroidal anti-inflammatory drugs Steroidal anti-inflammatory drugs steroidal anti-inflammatory	Enteral Systemic Systemic local Oral Sublingual Topic parenteral

**Table 2:** Search strategies in each database. Truncation operators (*) and Position operators (“)

Database	Search Strategy
Web of Science	(Antibiotics or Antibiotic therapy or NSAIDs or Analgesic* or Nonsteroidal anti-inflammatory or NSAID* or Non-steroidal anti-inflammatory drugs or Steroid anti-inflammatory drugs or steroidal anti-inflammatory) and (enteral or systemic or local or oral or sublingual) Soft tissue infection, Acute maxillary osteomyelitis “Osteomyelitis AND Maxilla”, Bone necrosis of the jaws, (Osteomyelitis or “Chronic Osteomyelitis” or “Acute Osteomyelitis” or Osteomyelitis or “Chronic Osteomyelitis” or “Acute Osteomyelitis”) and (Adult*) and (Treatment* pharmacological* or Drug* or medication* or treatment*)
Scopus	Acute osteomyelitis, (Osteomyelitis or “Chronic Osteomyelitis” or “Acute Osteomyelitis” or Osteomyelitis or “Chronic Osteomyelitis” or “Acute Osteomyelitis”) and (Adult*) and (Treatment* pharmacological* or Drug* or medication* or treatment*) and (Antibiotics or Antibiotic therapy or NSAIDs or Analgesic* or Nonsteroidal anti-inflammatory or NSAID* or Non-steroidal anti-inflammatory drugs or Steroidal anti-inflammatory drugs or steroidal anti-inflammatory) and (enteral or systemic or local or oral or sublingual), Bone necrosis of the jaws,
PubMed	(Osteomyelitis or “Chronic Osteomyelitis” or “Acute Osteomyelitis” or Osteomyelitis or “Chronic Osteomyelitis” or “Acute Osteomyelitis”) and (Adult*) and (Treatment* pharmacologic* or Drug* or medication* or treatment*) and (Antibiotics or Antibiotic therapy or NSAIDs or Analgesic* or Nonsteroidal anti-inflammatory or NSAID* or Non-inflammatory steroids or Anti-inflammatory steroids or steroidal anti-inflammatory) and (enteral or systemic or local or oral or sublingual), Sequestrectomy AND Saucerization, Bone necrosis of the jaws, “Osteomyelitis AND Maxilla”, Acute maxillary osteomyelitis.

**Table 3:** Eligibility criteria for inclusion and exclusion.

Inclusion Criteria	Exclusion Criteria
All participants were adults (≥18 years) diagnosed with acute or chronic osteomyelitis, with no previous clinical disorder, with no age, sex, or ethnicity restrictions.	Patients with bone infections of non-microbial origin or those who have not been specifically diagnosed with osteomyelitis by the referring dentist.
The intervention had to be antibiotic therapy in combination with nonsteroidal anti-inflammatory drugs (NSAIDs) or steroid anti-inflammatory drugs (SAIDs) administered before dental intervention	Studies with non-controlled designs or systematic review studies.
Comparisons were made between administration of medication before dental intervention compared with administration after dental intervention	Interventions that do not evaluate the combination of antibiotic therapy with NSAIDs/SAIDs
VAS measures, presence or absence of adverse drug reactions, were provided.	Articles in languages other than English, Spanish or Portuguese.
Reported statistical data allowed calculation of effect sizes.	

**Table 4:** Characteristics of included studies.

Author	Gender and Age of the Patient	Diagnosis	Pharmacological Treatment (Dose, Time)	Route of Administration	Follow-Up (Time Months)
Kudva et al., 2019 [27]	32-year-old male.	Recurrent mandibular osteomyelitis	Antibiotics Augmentin 125 mg, three times a day for one week) Clindamycin (300 mg, 2 times a day for one week) NSAIDs (without specification of NSAID used, three weeks) Glucocorticoid Prednisolone (40–60 mg, once daily for one week)	Antibiotics: IV acute phase, oral maintenance NSAIDs Oral Glucocorticoids: Oral	Yes (3 months)
Lambade et al., 2013, [28]	35-year-old female.	Chronic suppurative osteomyelitis of the mandibular condyle	Antibiotics Amoxicillin/clavulanate potassium (125 mg, three times a day) Metronidazole (400 mg, three times a day) NSAIDs Diclofenac sodium (50 mg, twice daily) for 7 days	Antibiotics: IV acute phase, chronic phase, oral maintenance NSAIDs: Oral Glucocorticoids: Oral	Yes (18 months)
Roldán et al., 2001, [29]	26-year-old male.	SAPHO syndrome (synovitis, acne, pustulosis, hyperostosis and osteitis)	Antibiotics Clindamycin (two years) Amoxicillin-clavulanate (500 mg/125 mg, 3 times a day for 7 days). Minocycline (50 mg, twice daily) Isotretinoin (10 mg, twice daily). Glucocorticoid Prednisolone (5 mg, once daily, three years)	Antibiotics: IV acute phase, chronic phase, oral maintenance NSAIDs: Oral Glucocorticoids Oral	Yes (3 years)
Holden et al., 2005, [24]	27-year-old female.	Presumptive diagnosis of osteomyelitis	Antibiotics Vancomycin, Meropenem, Doxycycline, Ciprofloxacin and Azithromycin (no dose specification) Glucocorticoid Methylprednisolone 1 g for two consecutive days (without specifying the time period)	Antibiotics: IV acute phase, oral maintenance NSAIDs: Oral Glucocorticoids Oral	Yes (3 years)

## Supplementary Materials

The following supporting information can be downloaded at: https://www.mdpi.com/article/10.3390/medicina61030499/s1, Table S1. Summary of Included Case Studies Details of the included case reports, including patient demographics, diagnosis, pharmacological treatment, administration routes, and follow-up outcomes. Table S2. Search Strategy Details. A breakdown of the specific search strategies used in Web of Science, Scopus, and PubMed, including search queries and Boolean operators. Figure S1. PRISMA Flowchart of Study Selection. A visual representation of the study selection process, including identification, screening, eligibility assessment, and final inclusion. Statistical Reliability Analysis. Table S3. Statistical Measures Cohen’s Kappa Coefficient (Inter-rater Agreement: 0.909) and Intraclass Correlation Coefficient (ICC: 1.0) for assessing inter-coder reliability.

## Appendix A

### Appendix A.2. CASP Checklist

CASP Checklist: 10 questions to help you make sense of a Systematic Review

How to use this appraisal tool: The following broad issues need to be considered when appraising a systematic review study.

Are the results of the study valid? (Section A)

What are the results? (Section B)

Will the results help locally? (Section C)

The 10 questions on the following pages are designed to help you think about these issues systematically. The first two questions are screening questions and have be answered quickly. If the answer to both is “yes”, it is worth proceeding with the remaining questions. There is some degree of overlap between the questions, and you are asked to answer with a “yes”, “no” or “can’t tell” to most of the questions. A number of italicized prompts are given after each question. These are designed to remind you why the question is important. Record your reasons for your answers in the spaces provided.

These checklists were designed to be used as educational pedagogical tools and part of a workshop setting; therefore, we do not suggest a scoring system. The core CASP checklists (randomized controlled trial and systematic review) were based on JAMA ’Users’ guides to the medical literature 1994 (adapted from Guyatt GH, Sackett DL, and Cook DJ), and piloted with health care practitioners.

For each new checklist, a group of experts were assembled to develop and pilot the checklist and the workshop format with which it would be used. Over the years, overall adjustments have been made to the format; however, to a recent survey of the checklist, users reiterated that the basic format continues to be useful and appropriate.

Referencing: We recommend using the Harvard style citation, i.e., Critical Appraisal Skills Programme (2018). CASP (insert yam of checklist i.e., Systematic Review) Checklist. [online]. OAP Ltd 59, Lakeside, Oxford, OX2 8JQ. info@casp-uk.net. Critical Appraisal Skills Programme (CASP), 2018. CASP Systematic Review Checklist. [online]. Available at: http://www.casp-uk.net (Accessed on 3 January 2025).

©CASP this work is licensed under the Creative Commons Attribution—Non-Commercial- Share TO like.

Critical Appraisal Skills Program (CASP) part of OAP Ltd. www.casp-uk.net

Paper for appraisal and reference:

1. Did the review address a clearly focused question?

Yes

Can’t Tell

No

HINT: An issue dog be ’focused’ In terms of

- the population studied;
- the intervention given;
- the outcome considered.

2. Did the authors look for the right type of papers?

Yes

Can’t Tell

No

HINT: ’The best sort of studies’ would

- address the reviews question;
- have an appropriate study design (usually RCTs for papers evaluating interventions).

3. Do you think all the important, relevant studies were included?

Yes

Can’t Tell

No

HINT: Look for

- which bibliographic databases are used;
- follow-ups from reference lists;
- staff contact with experts;
- unpublished as well as published studies;
- non-English language studies.

4. Did the review’s authors do enough to assess the quality of the included studies?

Yes

Can’t Tell

No

HINT: The authors need to consider the rigor of the studies they have identified. Lack of rigor may affect the studies’ results (“All that glitters is not gold” Merchant of Venice—Act II Scene 7)

5. If the results of the review have been combined, was it reasonable to do so?

Yes

Can’t Tell

No

HINT: Consider whether

- the results were similar from study to study;
- the results of all the included studies are clearly displayed;
- the results of different studies are similar;
- the reasons for any variations in results are discussed.

6. What are the overall results of the review?

HINT: Consider

- if you are clear about the reviews’ ‘bottom line’ results;
- what these are (numerically if appropriate);
- how the results were expressed (NNT, odds ratio, etc.).

7. How precise are the results? HINT: Look at the confidence intervals, if given

8. Can the results be applied to the local population?

Yes

Can’t Tell

No

HINT: Consider whether

- the patients covered by the review could be sufficiently different to your population to cause concern;
- your local setting is likely to differ much from that of the review.

9. Were all important outcomes considered?

Yes

Can’t Tell

No

HINT: Consider whether

- there is other information you would like to have seen.

10. Are the benefits worth the harms and costs?

Yes

Can’t Tell

No

HINT: Consider, even if this is not addressed by the review; what do you think?
