# Supplementary material for: Effectiveness and Safety of Antibiotic Therapy Combined with NSAIDs or SAIDs in Osteomyelitis of the Oral and Maxillofacial Region: A Systematic Review
Source: Medicina (Kaunas). 2025 Mar 13;61(3):499. doi: 10.3390/medicina61030499 (PMC11943866; doi:10.3390/medicina61030499)
Supplement: Supplementary file 1 [file medicina-61-00499-s001.zip › medicina-3458928-supplementary.pdf]

Supplementary Materials for:

Effectiveness and Safety of Antibiotic Therapy Combined with NSAIDs or SAIDs in Osteomyelitis of the Oral and Maxillofacial Region: A Systematic Review

Authors:

Heilyn Joanna Nils*, Cristina Arce Recatalá, Cosimo Galletti, and Javier Flores-Frail

1. Overview

This supplementary file provides additional methodological details, extended data tables, and complementary figures that support the findings in the main article.

2. Additional Data Tables

Table S1. Summary of Included Case Studies

Author	Age & Gender	Diagnosis	Treatment (Dose, Duration)	Administration Route	Outcome & Follow-up
Kudva et al. (2019)	32-year-old Male	Recurrent Mandibular Osteomyelitis	Augmentin (125 mg, 3x/day for 1 week), Clindamycin (300 mg, 2x/day for 1 week), NSAIDs (unspecified, 3 weeks), Prednisolone (40-60 mg, 1x/day for 1 week)	IV (acute phase), Oral (maintenance)	Stabilization of infection and pain control (3 months follow-up)
Lambade et al. (2013)	35-year-old Female	Chronic Suppurative Osteomyelitis	Amoxicillin/Clavulanate (125 mg, 3x/day), Metronidazole (400 mg, 3x/day), Diclofenac Sodium (50 mg, 2x/day for 7 days)	IV (acute), Oral (maintenance)	No recurrence, stable occlusion (18 months follow-up)
Roldán et al. (2001)	26-year-old Male	SAPHO Syndrome	Clindamycin (2 years), Amoxicillin-Clavulanate (500/125 mg, 3x/day for 7 days), Minocycline (50 mg, 2x/day), Isotretinoin (10 mg, 2x/day), Prednisolone (5 mg, 1x/day for 3 years)	IV (acute phase), Oral (chronic)	Partial resolution, persistent systemic symptoms (3 years follow-up)
Holden et al. (2005)	27-year-old Female	Chronic Multifocal Osteomyelitis	Vancomycin, Meropenem, Doxycycline, Ciprofloxacin, Azithromycin (unspecified doses), Methylprednisolone (1 g for 2 days)	IV (acute), Oral (maintenance)	Significant reduction in inflammatory markers (3 years follow-up)

Table S2. Search Strategy Details

Database	Search Query Used
Web of Science	("Antibiotics" OR "Antibiotic therapy" OR "NSAIDs" OR "Steroid anti-inflammatory drugs") AND ("osteomyelitis" OR "chronic osteomyelitis" OR "acute osteomyelitis") AND ("oral" OR "parenteral" OR "IV")
Scopus	("Osteomyelitis" AND "Treatment* pharmacologic*") AND ("Antibiotics" AND "NSAIDs" OR "SAIDs")
PubMed	("Osteomyelitis AND Maxilla" OR "Bone necrosis of the jaws") AND ("Clindamycin OR Amoxicillin-Clavulanate")

3. Extended Methodology

Search and Selection Process

- A comprehensive search was conducted across Web of Science, Scopus, and PubMed between July 2024 and November 2024.
- The inclusion criteria were randomized controlled trials (RCTs), cohort studies, and case-control studies investigating antibiotic therapy combined with NSAIDs or SAIDs for osteomyelitis.
- Studies without clear pharmacological regimens were excluded.

Data Extraction and Risk of Bias Assessment

- The **Critical Appraisal Skills Programme (CASP)** tool was used to assess the quality of included studies.
- Inter-coder reliability was measured using **Cohen's kappa coefficient (κ = 0.909)** and **intraclass correlation coefficient (ICC = 1.0)**.

4. Additional Figures

Figure S1. PRISMA Flowchart of Study Selection

(Illustration of the study identification, screening, eligibility, and inclusion process)

5. Statistical Reliability Analysis

Statistic	Value
Cohen's Kappa (Inter-rater Agreement)	0.909
Intraclass Correlation Coefficient (ICC)	1.0
Number of Studies Included	4

6. Additional Discussion Points

Safety Considerations in NSAID/SAID Use

- **NSAID Risks:** Gastrointestinal bleeding, renal dysfunction, potential inhibition of bone healing.
- **SAID Risks:** Immunosuppression, osteoporosis, metabolic complications.
- **Long-term Use Considerations:** Requires careful monitoring, especially in chronic osteomyelitis cases.

Future Research Directions

- Conduct **randomized controlled trials** (RCTs) to quantify the comparative efficacy of NSAID/SAID therapy.
- Investigate the role of **genetic markers** in osteomyelitis susceptibility and response to anti-inflammatory co-therapy.
- Explore **alternative immunomodulatory treatments** such as **bisphosphonates and TNF-α inhibitors**

7. References

(Additional references supporting the supplementary materials, if applicable, formatted in the journal's style)
